# Comorbid anxiety and depression among women receiving care for breast cancer: analysis of prevalence and associated factors

**DOI:** 10.4314/ahs.v22i3.19

**Published:** 2022-09

**Authors:** Nuworza Kugbey

**Affiliations:** Department of General Studies, University of Environment and Sustainable Development, Somanya, Ghana

**Keywords:** Breast cancer, depression, anxiety, doctor-patient relationship, shared decision making, Ghana

## Abstract

**Background:**

Living with breast cancer has been associated with increased risk for common mental health problems including depression and anxiety. However, the prevalence of comorbid anxiety and depression (CAD) and their associated factors have received little attention especially in low- and middle-income countries (LMICs) including Ghana.

**Objectives:**

This study examined the prevalence of CAD and its correlates in the context of breast cancer.

**Methods:**

Participants were 205 women receiving care for breast cancer at a Tertiary Hospital in Ghana. The Hospital Anxiety and Depression Scale (HADS) and socio-demographic questionnaires were administered to the participants.

**Results:**

Findings from the study showed that the prevalence of CAD, anxiety and depression was 29.4%, 48.5% and 37.3% respectively. CAD was significantly predicted by patients' English language reading ability, shared decision making and good doctor-patient relationship. Anxiety was significantly predicted by shared decision making and good doctor-patient relationship whereas depression was significantly predicted educational status, patients' English language reading ability, shared decision making and good doctor-patient relationship.

**Conclusion:**

The findings suggest relatively high prevalence of comorbid anxiety and depression which could negatively impact breast cancer treatment outcomes and therefore, improved interpersonal relationships between doctors and their patients as well as literacy skills are warranted.

## Introduction

Breast cancer is of the leading cancer types diagnosed among women and contributes to major disease burden^1^. Global mortality and morbidity rates contribute to substantial loss to the individual and society as a whole. However, advances in treatment strategies and preventive measures have led to improved survival chances. The treatment strategies have their associated complications ranging from physical, psychosocial, economic to spiritual distortions^2^, ^3^. For example, it has been reported that cancer patients experience physical health complications, psychological problems, social problems and economic challenges^4^, ^5^.

Mental health problems have been reported among breast cancer patients with the most prevalent ones being anxiety and depression^6^, ^7^. For instance, a systematic review revealed a global prevalence of depression among breast cancer patients to be 32.2% in studies from 30 countries^6^. A recent systematic review reported 41% prevalence of anxiety among women living with breast cancer^7^. These high rates of anxiety and depression pose a significant challenge to breast cancer management as the presence of anxiety and depression could interfere with treatment and overall health outcomes. Evidence in the oncology literature suggests that increased anxiety and depression levels among women living with breast cancer are predictive of poor quality of life and overall health outcomes^8^.

Some important socio-demographic and health system related factors are reported to be significantly associated with the presence of anxiety and depression among women living with breast cancer. Women's socio-demographic characteristics such as age, education, marital status, religions, employment status and incomes have been found to be significantly associated with the presence of anxiety and depression^9^–^11^. It has also been reported that the doctor- patient relationship and patients' involvement in their healthcare decision making contribute to the overall health and wellbeing including improved quality of life^12^. Patients' involvement and good relationships with their healthcare providers may serve to lessen the uncertainties that surround breast cancer and its treatment outcomes. The current study seeks to examine the prevalence of comorbid anxiety and depression (CAD), depression and anxiety among women living with breast cancer as most of the previous studies focused on either anxiety or depression in isolation. Co-occurrence of anxiety and depression in breast cancer patients may worsen their plight as the symptoms of these common mental health problems negatively affect treatment outcomes. The associated factors of CAD have also been examined to highlight the key risk and protective factors of the probability of experiencing co-occurrence of anxiety and depression among women living with breast cancer.

## Methods

### Participants and research design

Two hundred and five (205) womenreceiving care for breast cancer at the Korle-Bu Teaching Hospital were sampled. The participants had an average age of 52.49 years with majority being married (67.8%). About 90% of the participants identified as Christians and majority were employed (61%). A cross-sectional survey design was used as the main study design as a cross-section of the women receiving care for breast cancer was sampled for this study. Ethical clearance was sought from the Institutional Review Board of the Korle-Bu Teaching Hospital (KBTH-IRB/00035/2016) and all ethical guidelines were strictly adhered to in the study.

### Measures

Depression and anxiety were measured with the 14-item Hospital depression and anxiety scale^13^. This questionnaire has two sub-scales with 7-items each measuring depression and anxiety respectively with a 4-point Likert scale (0–3). A cut-off score of 8 and above out of 21 was classified as a case of depression and anxiety.

Shared decision making was measured with a single item (“Do you feel that you have been involved by your doctors/nurses in your treatment decision making?”) with a Yes/No response. Doctor-patient relationship was measured with the Doctor-Patien Relationship Questionnaire^14^ consisting of 9 items. The total score ranged between 9 and 45 and the mean score was used as a cut-off for good vs. poor doctor-patient relationship.

The other socio-demographic variables were assessed with a demographic questionnaire consisting of age, duration of illness, treatment, English language ability, Religion, comorbidities among others.

### Data Analysis

Descriptive statistics such as frequencies and means were used to summarize the data. Bivariate and multivariate associations between the correlates and the outcome variables (CAD, Anxiety and Depression) were done using chi-square and logistic regression (Unadjusted and Adjusted Odd Ratios) at the 0.05 level of significance.

## Results

Results from the analysis showed prevalence rates of 29.4%, 48.5% and 37.3% of CAD, anxiety and depression respectively. From the chi-square Table 1, it was observed that education status (χ^2^ = 3.739, p = .05), English language reading ability (χ^2^ = 7.218, p = .01), shared decision making (χ^2^ = 18.314, p < .01) and doctor-patient relationship (χ^2^ = 12.152, p < .01) were significantly associated with the presence of CAD among the participants.

**Table 1:** Bivariate associations between socio-demographic characteristics and CAD in breast cancer

Variables		Sample (205)	CAD	χ^2^	ρ
Age (195)^a^					
	Below 50years	44.1%	(26/88) 29.5%	.003	.96
	50years+	55.9%	(32/107) 29.9%		
Marital status (197)^a^					
	Married	67.8%	(38/137) 27.7%	.629	.53
	Unmarried	32.2%	(20/60) 33.3%		
Education (197)^a^					
	Formal	89.3%	(48/176) 27.3%	3.739	.05*
	No formal	10.7%	(10/21) 47.6%		
Employment (196)^a^					
	Employed	61.3%	(33/122) 27.0%	1.003	.40
	Unemployed	38.7%	(25/74) 33.8%		
Average monthly income (197)^a^					
	Less than $100	48.3%	(28/95) 29.5%	.001	.99
	$100 or more	51.7%	(30/102) 29.4%		
Religion (197)^a^					
	Christian	89.8%	(51/176) 29.0%	.171	.87
	Non-Christian	10.2%	(7/21) 33.3%		
Comorbid medical condition (194)^a^					
	Yes	38.1%	(20/75) 26.7%	.434	.62
	No	61.9%	(37/119) 31.1%		
English reading ability (196)^a^					
	Yes	70.1%	(33/138) 23.9%	7.218	.01**
	No	29.9%	(25/58) 43.1%		
Shared decision making (196)^a^					
	Yes	84.2%	(38/164) 23.2%	18.314	<.01***
	No	15.85	(19/31) 61.3%		
Doctor-patient relationship (196)^a^					
	Poor	58.8%	(45/115) 39.1%	12.152	<.01***
	Good	41.2%	(13/81) 16.0%		

CAD= Comorbid anxiety and depression

a missing values observed

* significant at .05)

Results from Table 2 showed that only shared decision making (χ^2^ = 5.345, p =02) and doctor-patient relationship (χ^2^ = 10.051, p < .01) were significantly associated with the presence of anxiety among the participants.

**Table 2:** Bivariate associations between socio-demographic characteristics and anxiety in breast cancer

Variables		Anxiety	χ^2^	ρ
Age (198)^a^				
	Below 50years	(48/88) 54.5%	1.956	.21
	50years+	(49/110) 44.5%		
Marital status (200)^a^				
	Married	(67/137) 48.9%	.029	.99
	Unmarried	(30/63) 47.6%		
Education (200)^a^				
	Formal	(84/178) 47.2%	1.110	.41
	No formal	(13/22) 59.1%		
Employment (199)^a^				
	Employed	(60/125) 48.0%	.074	.90
	Unemployed	(37/74) 50.0%		
Average monthly income (200)^a^				
	Less than $100	(47/97) 48.5%	.001	.99
	$100 or more	(50/103) 48.5%		
Religion (200)^a^				
	Christian	(85/179) 47.5%	.702	.54
	Non-Christian	(12/21) 57.1%		
Comorbid medical condition (197)^a^				
	Yes	(37/75) 49.3%	.060	.92
	No	(58/122) 47.5%		
English reading ability (199)^a^				
	Yes	(63/139) 45.3%	2.158	.19
	No	(34/60) 56.7%		
Shared decision making (198)^a^				
	Yes	(74/166) 44.6%	5.345	.02*
	No	(22/32) 68.8%		
Doctor-patient relationship (199)^a^				
	Poor	(69/118) 58.5%	10.051	<.01***
	Good	(28/81) 34.6%		

a missing values observed

* significant at .05

It was observed from Table 3 that education status (χ^2^ = 4.946, p = .03), English language reading ability (χ^2^ = 13.272, p < .01), shared decision making (χ^2^ = 24.198, p < .01) and doctor-patient relationship (χ^2^ = 8.758, p < .01) were significantly associated with the presence of depression among the participants.

**Table 3:** Bivaria te associations between socio-demographic characteristics and depression in breast cancer

Variables		Depression	χ^2^	ρ
Age (198)^a^				
	Below 50years	(32/88) 36.4%	.013	.91
	50years+	(42/110) 38.2%		
Marital status (201)^a^				
	Married	(48/139) 34.5%	1.130	.29
	Unmarried	(27/62) 43.5%		
Education (201)^a^				
	Formal	(62/180) 34.4%	4.946	.03*
	No formal	(13/21) 61.9%		
Employment (200)^a^				
	Employed	(42/122) 34.4%	.947	.33
	Unemployed	(33/78) 42.3%		
Average monthly income (201)^a^				
	Less than $100	(36/97) 37.1%	.003	.96
	$100 or more	(39/104) 37.5%		
Religion (201)^a^				
	Christian	(65/180) 36.1%	.630	.42
	Non-Christian	(10/21) 47.6%		
Comorbid medical condition (198)^a^				
	Yes	(28/77) 36.4%	.007	.93
	No	(46/121) 38.0%		
English reading ability (200)^a^				
	Yes	(41/141) 29.1%	13.272	<.01**
	No	(34/59) 57.6%		
Shared decision making (199)^a^				
	Yes	(49/168) 29.2%	24.198	<.01**
	No	(24/31) 77.4%		
Doctor-patient relationship (200)^a^				
	Poor	(54/116) 46.6%	8.758	<.01***
	Good	(21/84) 25.0%		
	

a missing values observed

* significant at .05)

**Table 4:** Multivariate associations between socio-demographic factors and CAD in breast cancer

**Table 4:** Multivariate associations between socio-demographic factors and CAD in breast cancer

Variables		CAD		Anxiety		Depression	
		OR	AOR	OR	AOR	OR	AOR
Age in years							
	Below 50years	1	1	1	1	1	1
	50years+	.98 (.53 –1.82)	.92 (.42–2.00)	.67 (.38–1.18)	.59 (.30–1.17)	1.08 (.61–1.93)	.84 (.39–1.83)
Marital status							
	Married	1	1	1	1	1	1
	Unmarried	.77 (.34–1.48)	.68 (.31–1.51)	.95 (.52–1.73)	.93 (.47–1.85)	.68 (.37–1.26)	.60 (.27–1.29)
Education							
	No formal	1	1	1	1	1	1
	Formal	.41 (.17–1.00)	.84 (.24–2.96)	.62 (.25–1.52)	1.04 (.32–3.38)	.32* (.13–.82)	.72 (.20–2.58)
Employment status							
	Unemployed	1	1	1	1	1	1
	Employed	.73 (.34–1.34)	.70 (.31–1.57)	.92 (.52–1.64)	.83 (.41–1.68)	.72 (.40–1.28)	.63 (.29–1.39)
Average monthly income (USD$)							
	Less than $100	1	1	1	1	1	1
	$100 or more	.99 (.54–1.84)	1.39 (.63–3.07)	1.00 (.58–1.75)	1.12 (.57–2.20)	1.02 (.57–1.80)	1.59 (.74–3.42)
Religion							
	Non-Christian	1	1	1	1	1	1
	Christian	.82 (.31–2.14)	1.24 (.36–4.35)	.68 (.27–1.69)	.84 (.28–2.48)	.62 (.25–1.54)	.96 (.29–1.39)

Comorbid medical condition							
	No	1	1	1	1	1	1
	Yes	.81 (.42–1.53)	.75 (.34–1.65)	1.07 (.60–1.91)	1.21 (.62–2.38)	.93 (.52–1.68)	1.04 (.49–2.21)

English reading ability							
	No	1	1	1	1	1	1
	Yes	.42** (.22–.80)	.31** (.12–.77)	.63 (.34–1.17)	.47 (.21–1.08)	.30*** (.16–.57)	.25** (.10–.62)
Shared decision making							
	No	1	1	1	1	1	1
	Yes	.19*** (.09–.43)	.26** (.10–.64)	.37* (.16–.82)	.48 (.19–1.19)	.12*** (.05–.30)	.12** (.04–.34)
Doctor-patient relationship							
	Poor	1	1	1	1	1	1
	Good	.30** (.15–.60)	.36 (.16–.80)	.38** (.21–.67)	.41** (.22–.80)	.38** (.21–.71)	.52 (.25–1.10)

Results from logistic regression analysis (Table 4) showed that participants with English language reading ability were 58% less likely to report CAD, participants who felt involved in their treatment decision making were 81% less likely to experience CAD and participants who reported a good doctor-patient relationship were 70% less likely to experience CAD in the unadjusted model but only shared decision making and doctor-patient relationship remained significant in the adjusted model. For anxiety, it was observed that participants who felt involved in their treatment decision making were 63% less likely to experience anxiety and participants who reported a good doctor-patient relationship were 62% less likely to experience anxiety in the unadjusted model but only doctor-patient relationship remained significant in the adjusted model. For depression, it was observed that participants with formal education were 68% less likely to experience depression, participants with English language reading ability were 70% less likely to report depression, participants who felt involved in their treatment decision making were 88% less likely to experience depression and participants who reported a good doctor-patient relationship were 62% less likely to experience depression in the unadjusted model but only English language reading ability and shared decision making remained significant in the adjusted model.

## Discussion

Findings from the study showed relatively high prevalence of common mental health problems among women receiving care for breast cancer with prevalence rates of 29.4%, 48.5% and 37.3% of CAD, anxiety and depression respectively. These high rates of common mental health problems reported in this study are consistent with previous literature on depression and anxiety among women living with breast cancer^6^,^7^. However, the prevalence of CAD in the study was lower than 87% anxiety-depressive syndrome (ADS) reported among breast cancer patients in Morocco^4^. The disparities could be due to differences in the socio-economic and other prevailing circumstances in the two countries. The Moroccan study period coincided with the COVID-19 which might explain some of the variations in the prevalence rates.

Evidence from this study showed that educational status, English language reading ability, shared decision making and good doctor-patient relationship were significant predictors of CAD, anxiety and depression. Participnts with English reading ability, involved by the healthcare providers in their treatment decisions and good doctor-patient relationship were less likely to experience comorbid anxiety and depression. The findings showed similar predictors for the common mental problems except for depression with formal education being a protective factor against the experience of depression. The role of doctor-patient relationship and shared decision making in health outcomes has been reported in some studies among women living with breast cancer^12^, ^15^, ^16^.

## Limitations

This study is limited by the relatively small sample size used and data from only one oncology centre which may not reflect what pertains in other parts of the country. Despite these limitations, this study is an exploratory one which is likely to serve as basis for a multicentre study exploring mental health issues and their associated factors on a large scale.

## Conclusion

This study highlights the burden of common mental health problems especially comorbid anxiety and depression (CAD) among women receiving medical care for breast. There is the need for regular psychological screening as part of routine oncology care using brief mental health assessment tools such Distress Thermometer (DT) to inform treatment decisions and appropriate referrals.
